# Mental health of Tunisian women during the first wave of COVID-19 pandemic

**DOI:** 10.1192/j.eurpsy.2021.2205

**Published:** 2021-08-13

**Authors:** S. Sediri, Y. Zgueb, A. Aissa, U. Ouali, F. Nacef

**Affiliations:** Psychiatry A Department, Razi Hospital, Manouba, Tunisia

**Keywords:** Anxiety, Depression, stress, coronavirus

## Abstract

**Introduction:**

The coronavirus disease 2019 (COVID-19) pandemic created a situation of general distress. Although the focus has been initially more on the physical health during the pandemic, mental health concerns linked to the lockdown have quickly risen.

**Objectives:**

The aim of this study was to evaluate the impact of the first wave of COVID-19 pandemic on Tunisian women’s mental health.

**Methods:**

An online survey was conducted during the first wave of COVID-19 pandemic using the Depression Anxiety and Stress Scales (DASS-21). We also collected sociodemographic information and mental health status.

**Results:**

A total of 751 women completed the questionnaire. More than half of the participants (57.3%) reported extremely severe distress symptoms, and 53.1% had extremely severe stress symptoms as per the DASS-21. Those who had a history of mental illness were found to have more severe symptoms of depression, anxiety and stress.

**Conclusions:**

As this study was the first one to evaluate the acute impact of COVID-19 on mental health in Tunisia, Arab world and in Africa, it may be a sound basis for developing an effective psychological intervention aimed at women in these regions.

**Disclosure:**

No significant relationships.

